# Transcriptomic Analysis of *Staphylococcus aureus* Under the Stress Condition Caused by *Litsea cubeba* L. Essential Oil via RNA Sequencing

**DOI:** 10.3389/fmicb.2020.01693

**Published:** 2020-09-08

**Authors:** Yunqiao Yang, Yunru Chen, Geyin Zhang, Junying Sun, Lei Guo, Mingsheng Jiang, Bingming Ou, Weiyu Zhang, Hongbin Si

**Affiliations:** ^1^College of Animal Sciences and Technology, Guangxi University, Nanning, China; ^2^College of Bioscience and Biotechnology, Yangzhou University, Yangzhou, China; ^3^College of Life Science, Zhaoqing University, Zhaoqing, China

**Keywords:** *Litsea cubeba* L., essential oil, *Staphylococcus aureus* 29213, staphyloxanthin, RNA sequencing

## Abstract

*Litsea cubeba* L. essential oil (LCEO) is a natural essential oil with considerable antimicrobial activity, and it can gradually replace some chemical additives in the food industry. However, the genetic evidences of stress response of bacteria under sub-lethal treatment with LCEO is limited. To this end, transcriptomic analysis of *Staphylococcus aureus* 29213 under a low concentration of LCEO was performed. Bacterial RNA samples were extracted from 1/4 MIC (0.07 μL/mL) of LCEO-treated and non-treated *S. aureus* 29213. The transcriptional results were obtained by RNA sequencing (RNA-Seq). After treated with LCEO of *S. aureus* 29213, 300, and 242 genes were significantly up and down-regulated. Up-regulated genes were mainly related to cell membrane (wall) stress stimulon including genes related to two-component regulatory system (*VraS*), histidine metabolism (*hisABCD* etc.) and L-lysine biosynthesis (*thrA, lysC, asd* etc.). Significant differences were also founded between LCEO-treated and non-treated groups in peptidoglycan biosynthesis related pathways. Down-regulated genes were related to nitrogen metabolism (*NarGHIJ* etc.), carotenoid biosynthesis (all) and pyruvate metabolism (*phdA, pflB, pdhC* etc.) of *S. aureus* 29213 in an LCEO-existing environment compared to the control. At the same time, we confirmed that LCEO can significantly affect the staphyloxanthin level of *S. aureus* 29213 for the first time, which is closely related to the redox state of *S. aureus* 29213. These evidences expanded the knowledge of stress response of *S. aureus* 29213 strain under sub-lethal concentration of LCEO.

## Introduction

*Litsea cubeba* L. (*L. cubeba*) is a plant of the *Lauraceae* family and distributed in many countries in East Asia, and it is mainly distributed in the Southern and western parts of China (Liu and Yang, 2012). All parts of the plant have a ginger-like smell (Ho et al., 2010). The fruits of *L. cubeba* are often used by people from Taiwan as a condiment for dishes (Liao et al., 2015). As an ethnobotanical material, *L. cubeba* has been used for headache, fatigue, chronic tracheitis and acute mastitis; the fresh leaves of it are grinded into a powder then used for skin problems, such as snake bite and furuncles (Chen et al., 2013; Nguyen et al., 2016). In the past decade, many studies focused on the pharmacological functions and the chemical compositions of *L. cubeba* essential oil (LCEO). The main component of *L. cubeba* in the fruit leaf, flower and twig oils is citral, 1,8-cineole, while in the stem oil there are limonene, citronellal, and citronellol (Ho et al., 2010). Research about the difference of main chemical composition in LCEO from various regions of China was also published (Si et al., 2012). The fruit oil of LC has been used for the flavor enhancer in cosmetics, cigarettes and food products (Wang et al., 2009; Liu and Yang, 2012). In some Chinese food factories, LCEO was used as food preservatives. Meanwhile, LCEO can be used as a material for citral (neral and geranial) refining in the market (Zhili et al., 2009). In pharmacological research, LCEO have been reported to have antioxidative (Hwang et al., 2005; She et al., 2019), anticancer [10,15] insecticidal (Seo et al., 2009) and antimicrobial activities (Wang and Liu, 2010; Liu and Yang, 2012).

*Staphylococcus aureus* (*S. aureus*) is a serious bacterial pathogen that causes infections (e.g., sepsis, meningitis, pneumonia, endocarditis, osteomyelitis, vomiting, nausea and abdominal cramping) and food poisoning in many animals and humans (Lowy, 1998; Argudin et al., 2010). Resent study has shown that the antibacterial ability of LCEO which was extracted in July on *S. aureus* was stronger than that of any other months (She et al., 2019). Research findings indicated the destructive effect of LCEO on the cell membrane of methicillin-resistant *S. aureus* (MRSA) caused the leakage of intracellular macromolecules in cell (Hu et al., 2019). However, the deep impact of LCEO on *S. aureus* has not been well studied.

In recent years, various molecular tools and bioinformatics techniques have been widely used for identifying and analyzing how antibacterial agent interact with bacteria more accuracy and efficiency (Park et al., 2019). Thanks to the next generation sequencing, transcriptome analysis plays a significant role in understanding the mechanism of microbe which was treated by an antibacterial agent (Delpech et al., 2015). The aim of this study is to elucidate the stress response of *S. aureus* 29213 strain under sub-lethal concentration of LCEO at transcriptome level by RNA-Seq method.

## Materials and Methods

### Oil and Bacterial Strains

*Litsea cubeba* L. essential oil was came from a local agency (Nanning, China) and the chemical compositions of LCEO was shown in Table 1. The LCEO in present work was extracted from the fruit of LC. As essential oils do not dissolve in water, LCEO, tween-80 and distilled water (volume ratio of 1: 1: 5) was mixed to disperse the LCEO in water and the hydrophile-lipophile balance number of this system was 15.

**TABLE 1:** Chemical compositions of LCEO.

Composition	Ratio (%)
α-Citral	38.28
β-Citral	29.29
Cinene	16.5348
Eudesmol	2.56
Citronellal	2.2383
Trans-Verbenol	2.06
Aromatic alcohol	1.86
β-Pinene	1.5104
Cis-Verbenol	1.3771
α-Vinyl acetate	1.0945
Geraniol	0.93
α-Pinene	0.7307
2-Methyl-2-Hexen-6-One	0.6994

*S. aureus* ATCC 29213 strain has the ability to form biofilm and to generate staphyloxanthin as well as it was often used in antimicrobial research (Musthafa and Voravuthikunchai, 2015; Sadrearhami et al., 2019; Singh et al., 2019). In this study, the *S. aureus* 29213 strain was provided by The First People’s Hospital of Nanning, China.

One milliliter *S. aureus* 29213 was taken out from the refrigerator (−80°C) then inoculated into tryptic soy broth (TSB) at 37°C for 36 h with 250 RPM shake, then 10 μL cultured solution was incubated into TSB at 37°C, 12 h with 250 RPM shake for viability recover. One microliter cultured solution was taken by inoculation hard loop to inoculate on a plate with tryptone soy agar (TSA) at 37°C for 24 h. A single colony was incubated in a tube with 3.5 mL of TSB at 37°C with shaking at 250 rpm for 8 h before subsequent trials.

### Determination of Minimum Inhibitory Concentrations (MICs)

Minimum Inhibitory Concentration values of LCEO on *S. aureus* 29213 strain was determined by the serial-dilution culture method.

TSB was used as the incubation medium. The LCEO solution was serially diluted by TSB. All tubes (13 × 100 mm) contained 1.75 mL LCEO solution and 1.75 mL of diluted *S. aureus* 29213 inoculum (approximately 10^4^ CFU/mL), with a final concentration of LCEO solution of 71.4, 35.7, 17.8, 8.9, 4.5, 2.2, 1.1, 0.56, 0.28, 0.014, 0.070, and 0.035 μL/mL. After incubation at 37°C for 24 h, MICs were measured by visual inspection of the turbidity of broth in tubes (Yang et al., 2020). That is to say, if the test tube was still clear and transparent (un-cloudy) after incubation, the cells cannot grow at that concentration and then the MIC value was obtained. Vancomycin was used as a positive anti-*S. aureus* control. This assay was carried out in triplicate.

### Preparation of Bacterial Samples for RNA-Seq

A conical flask (150 mL) with TSB (45 mL) was inoculated with fresh *S. aureus* 29213 strain culture (500 μL) which mentioned above and then incubated at 37 °C with 250 rpm shaking. Cell samples were taken for LCEO stimulation as the optical density value (OD600) at 0.8 (1 × 10^6^ CFU/mL) and subsequently, 3 samples of O_S29 group (O_S29_1, O_S29_2, O_S29_3) were treated with 1/4 MIC (0.07 μL/mL) of LCEO for 15 min. The control group (S29 group, 3 samples) was without incubated with LCEO and were also kept in the same conditions for 15 min.

### RNA Extraction and Purification, cDNA Library Construction for RNA-Seq, RNA-Seq, and Data Analysis

Total RNA was extracted from 6 bacterial samples (O_S29_1, O_S29_2, O_S29_3, S29_1, S29_2, S29_3) separately by a Total RNA Isolation System (Promega) according to the manufacturer’s protocol. The quality of the RNA samples was examined using the Agilent 2100 Bioanalyzer. Library construction and Illumina sequencing was performed by Novogene China. An RNA-seq analysis was performed according to the protocol recommended by the manufacturer (Illumina Inc.) (Qiao et al., 2016; Liu et al., 2019).

For data analysis, RNA-Seq reads were mapped to the reference genome of *S. aureus* Newman (NC_009641). Differentially expressed genes (DEGs) were identified by the edgeR package based on Genes with two criterions: |log2(fold change)| > 0.58499 (|fold change| >1.50003) and *p* < 0.05

### Quantitative Real-Time PCR

To ensure the accuracy of RNA-seq data, 8 DEGs were selected for quantitative real-time PCR (RT-PCR) verification and they were based on two standards: (a) Functional classification. The 8 DEGs were selected from different GO terms and KEGG pathways which were significantly enriched. (b) The value of fold change. 4 DEGs were up-regulated and the other 4 DEGs down-regulated and with |fold change| > 1.8. All experiments were performed in triplicate with at least three independent experiments.

Quantitative real-time PCR assay was carried out as described previously with some modifications (Park et al., 2019). The cDNA for qRT-PCR was synthesized using a quanti tect reverse transcription kit [Sangon Biotech (Shanghai) Co., Ltd.]. QRT-PCR was performed using specific primer pairs and iQTM SYBR Green supermix [Sangon Biotech (Shanghai) Co., Ltd.]. The primers were designed with the Primer-BLAST (NCBI) and purchased from Sangon Biotech (Shanghai) Co., Ltd., as shown in Table 2. The amplification and detection of PCR products were performed using CFX Connect^TM^ Real-Time System (Bio-Rad). The thermal cycling conditions were as follows. After activation of the polymerase and a DNA denaturation step at 95°C, 40 amplification cycles were performed with a denaturation step at 95°C followed by an annealing and extension step at 55°C. The cDNA values were normalized with the value of 16s rRNA, which was constant in different conditions (data not shown).

**TABLE 2:** Primer sequences used for quantitative real-time PCR.

Gene name	Primer sequence (5′- > 3′)	NCBI-Protein ID	Products (base pairs)
arcC	TACGCGGCACAAGGTAAGTT	BAF68803	127
	AAGCTTCGTATGCCTGCTCT		
crtM	TCGTAGAATCATGATGGCGCT	BAF68734	126
	TCAGCGTCCGTTTCAAACAT		
lrgB	TGGCATCGTATCATCGGAGG	BAF66469	133
	TCGCTGTAGTTGCTGCTTGA		
adh1	TGTGGCGTTTGTCATACCGA	BAF66849	149
	GCGATAGACACACGGTCTCC		
thrA	AATTCGGTGGTAGCTCCGTC	BAF67511	104
	ACCTGGAGCAGAAACGATAACA		
sdrC	CATGAAGCTAAAGCGGCAGA	BAF66795	139
	CTGCAGTTGCAGTTTGCGTAT		
hisD	GCCTGAACATGCGTCGATTC	BAF68848	131
	CATGACTTGGACCTGCAACG		
ldh1	TGGTGTTCCAGCAGTCATCA	BAF66448	93
	GCTGAATGTGCGAACTTGCT		

### Staphyloxanthin Levels Assay

Staphyloxanthin production was measured as described previously with some modifications (Singh et al., 2018). Briefly, 8 h *S. aureus* 29213 strain cultures were diluted 1:100 in tubes with 3.5 mL TSB at 250 RPM shaken and 37°C for 24 h with different lever of LCEO: 1/2 (0.14 μL/mL), 1/4 (0.07 μL/mL), and 1/8 (0.035 μL/mL) MIC. The control samples (without LCEO) were incubated in the same culture conditions. All the culture in tubes were centrifugated and then washed with distilled water for two times. Each tube was added into 2 mL methanol for the ultrasonic extraction of staphyloxanthin under 600 watt at 55°C for 20 min, and the supernatant with staphyloxanthin was obtained by centrifugation. The value of OD465 was measured as the biosynthesis level of staphyloxanthin. Three independent experiments were run in triplicate.

### Statistical Analysis of Quantitative Real-Time PCR and Staphyloxanthin Levels Assay

Statistical analysis was performed using SPSS (version 16.0, SPSS Inc., Chicago, IL, United States). Differences between groups were analyzed using one-way analysis of variance (ANOVA) followed by *post hoc* analysis using Tukey test. Differences were considered significant at *P* < 0.05.

## Results and Discussion

### General Features of the Transcriptome Profile

RNA-Seq generated total 9,494,858 to 11,617,170 clean reads from LCEO-treated (O_S29) and control (S29) cDNA libraries, respectively. These clean reads were mapped to the reference genome of *S. aureus* Newman (NC_009641) (Supplementary Data Sheet 1). RNA-seq data were available for further transcriptome analysis as the gene expression levels of three independent sample in each group were highly consistent. The expression level of genes were determined by average Per Kilobase of transcript per Million mapped reads (RPKM, full information of reads quantification) and these values showed that 2989 total genes were expressed in LCEO-treated and control groups.

The raw sequence data reported in this paper have been deposited in the Genome Sequence Archive (Genomics, Proteomics and Bioinformatics 2017) in BIG Data Center (Nucleic Acids Res 2019), Beijing Institute of Genomics (BIG), Chinese Academy of Sciences, under accession numbers CRA002405 that is publicly accessible at https://bigd.big.ac. cn/gsa.

### Differentially Expressed Genes in LCEO-Treated *S. aureus* 29213

**FIGURE 1:**
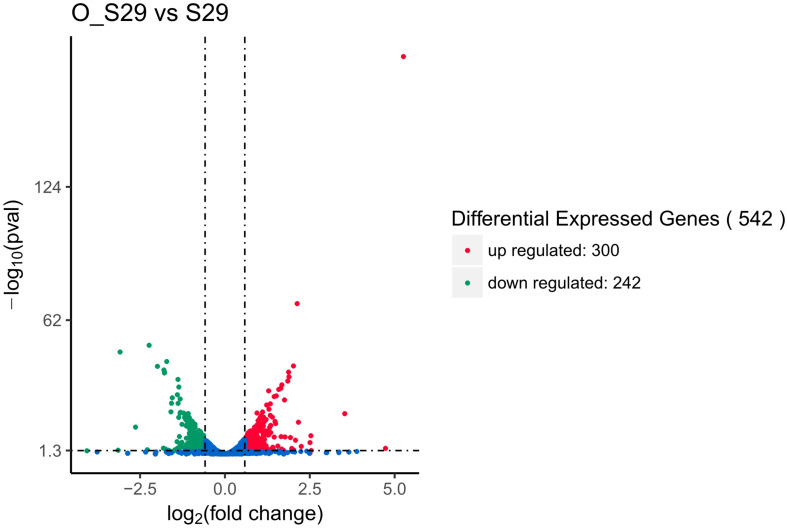
Differential expression level of LCEO-treated (O_S29) and non-treated *S. aureus* 29213 (S29) groups identified by |log2(fold change)| >0.58499 (|fold change| >1.50003) and *p* < 0.05.

Identified by adjusted |log2(fold change)| >0.58499 (|fold change| >1.50003) and *p* < 0.05, 542 DEGs of LCEO-treated group with 300 and 242 genes were up and down-regulated, respectively, compared to the control (Figure 1 and Supplementary Table 1). In the differentially expressed genes, 12 significantly up-regulated genes and 6 down-regulated genes showed |log2(fold change)| >2. According to the gene ID and gene name of the NCBI database, 12 up-regulated genes encode superantigen-like protein, ATP phosphoribosyltransferase regulatory subunit, ATP phosphoribosyltransferase catalytic subunit, histidinol dehydrogenase, imidazoleglycerol-phosphate dehydratase, fibrinogen-binding protein and 6 hypothetical proteins. The 6 down-regulated genes encode antiholin-like protein LrgB, murein hydrolase regulator LrgA, myosin-cross-reactive antigen and 3 hypothetical proteins.

### GO Functional Enrichment Analysis

**FIGURE 2:**
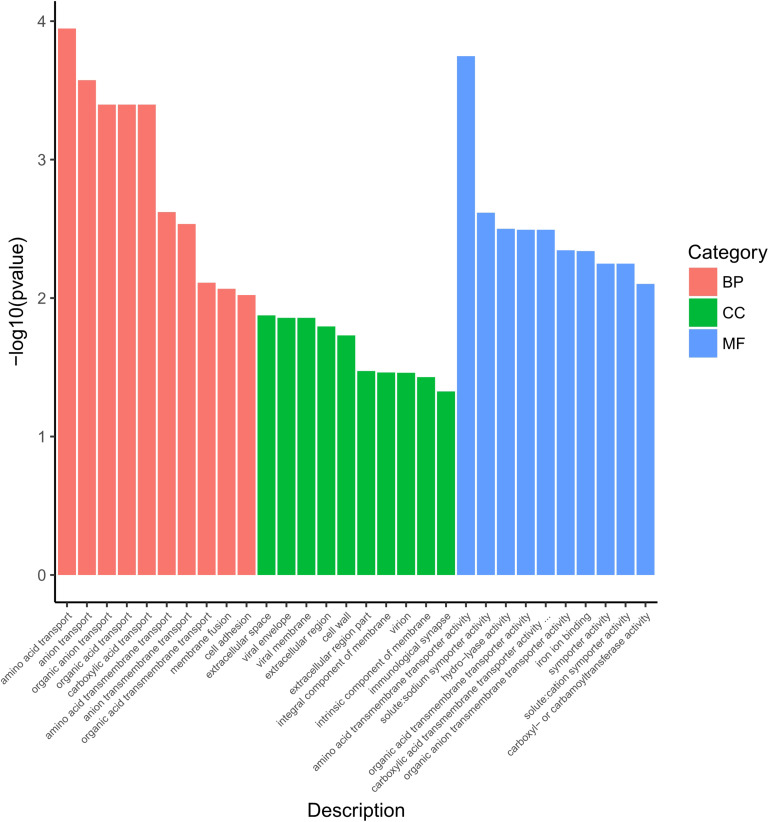
Significantly enriched GO terms of differentially expressed genes. BP, biological process; CC, cellular component; MF, molecular function.

The 542 DEGs were processed by Gene Ontology (GO) enrichment analysis for understanding the stress response at genetic level of *S. aureus* 29213 which was treated by 1/4 MIC LCEO. As shown in Supplementary Table 2, 910, 180, and 451 specific GO terms in biological process, cellular component and molecular function were reported, respectively. In Figure 2, 30 significantly enriched GO terms from three specific categories were showed.

Ten GO terms were enriched in the biological process, including amino acid transport, anion transport, organic anion transport, organic acid transport, carboxylic acid transport, amino acid transmembrane transport, anion transmembrane transport, organic acid transmembrane transport, membrane fusion, and cell adhesion.

Ten GO terms were enriched in the cellular component, including extracellular space, viral envelope, viral membrane, extracellular region, cell wall, extracellular region part, integral component of membrane, virion, intrinsic component of membrane, and immunological synapse.

In the category of molecular function, Ten GO terms (amino acid transmembrane transporter activity, solute:sodium symporter activity, hydro-lyase activity, organic anion transmembrane transporter activity, iron ion binding, symporter activity, organic acid transmembrane transporter activity, solute:cation symporter activity, carboxyl- or carbamoyltransferase activity, and carboxylic acid transmembrane transporter activity) were significantly enriched.

### KEGG Pathway Enrichment Analysis

**FIGURE 3:**
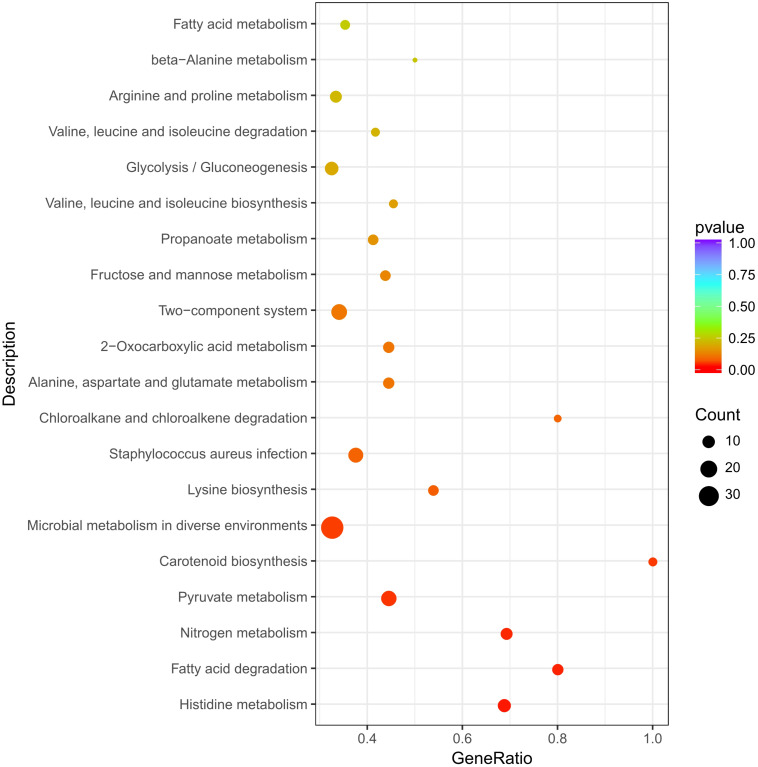
Significantly enriched KEGG pathways of differentially expressed genes.

The 542 DEGs were mapped to the KEGG database, and then analyzed by KEGG pathway enrichment analysis to understand the involved pathways (Supplementary Table 3). As shown in Figure 3, KEGG pathways: histidine metabolism, fatty acid degradation, nitrogen metabolism, pyruvate metabolism, carotenoid biosynthesis were significantly enriched.

### DEGs Involved in “Histidine Metabolism,” “Lysine Biosynthesis,” “Two-Component System” and “Peptidoglycan Biosynthesis” Pathways

Study has shown that the leakage of intracellular macromolecules was caused by the destructive effect of 0.5 mg/mL (1 MIC) LCEO on the cell membrane of MRSA (Hu et al., 2019). Actually, based on the transmission electron microscopy (TEM) micrographs provided by this reference above, the cell wall of the bacterium was also damaged by LCEO. Therefore, LCEO has the effect of disintegrating cell walls and membranes of *S. aureus*. In this experiment, 1/4 mic of LCEO also triggered cell wall (membranes) stress to *S. aureus* 29213.

**FIGURE 4:**
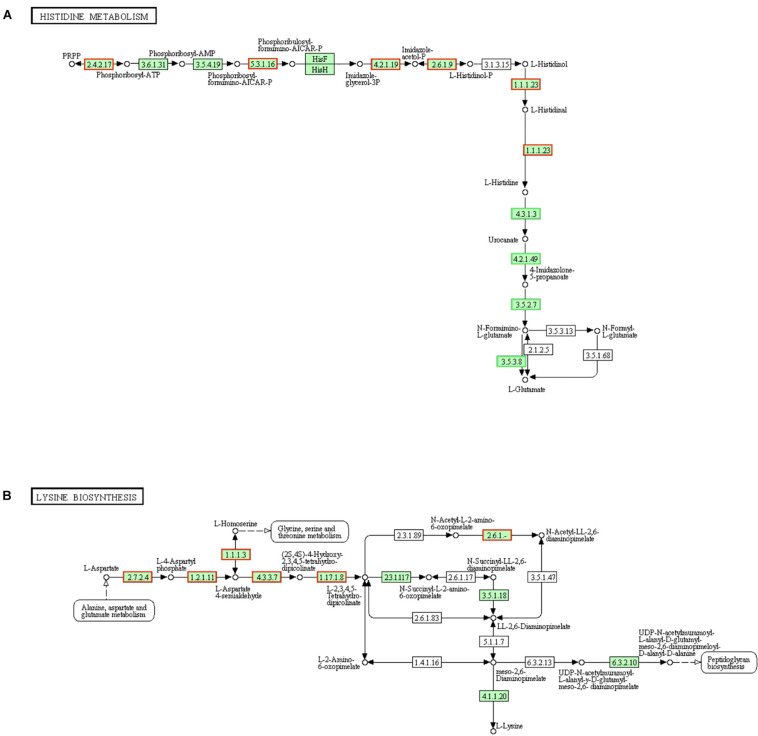
Significantly enriched KEGG pathway “Histidine metabolism” **(A)** and “Lysine biosynthesis” **(B)** (from KEGG database, green rectangles represent proved Organism-specific gene product and green frames represent down-regulation and red frames represent up-regulation).

Histidine protein kinases (HPKs) are a large family of signal-transduction enzymes. The typical HPK is a transmembrane receptor and it can autophosphorylate the conserved histidine residue of itself (Wolanin et al., 2002). Bacterial two-component signaling system allows organisms to perceive and respond to change in many different environmental conditions. Some signal-transduction were achieved by the sensor histidine kinase (HK) and phosphorylation response regulator (RR) of two-component system (Harms et al., 2001). Well, it is easy to know that histidine is urgently needed in the repair process once the cell wall (membranes) is damaged. There are ten steps of biosynthesis from PRPP to L-histidine, and the 3 key enzymes (hisB, hisC, and hisI) are the utmost importance in this histidine biosynthesis pathway of *S. aureus* (Henriksen et al., 2011). Here, in the histidine metabolism pathways, genes (encoding ATP phosphoribosyltransferase [EC:2.4.2.17], hisA [EC:5.3.1.16], hisB [EC:4.2.1.19], hisC [EC:2.6.1.9] and hisD [EC:1.1.1.23]) in LCEO treated *S. aureus* 29213 and were significantly up-regulated as comparison with the control. L-histidine can be metabolized into L-glutamate through a series of reactions. Simultaneously, 4 (all the) annotated organism-specific genes in these steps were also down-regulated. These results suggested that the histidine biosynthesis of *S. aureus* 29213 was increased under the stimulation of 1/4 MIC of LCEO (Figure 4A).

Like alanine and glutamate, L-lysine is not only used to synthesize proteins but also a component of peptidoglycan of cell wall in gram-positive bacteria. Meso-2,6-diaminopimelate can be utilized in the biosynthesis process of peptidoglycan and L-lysine (Grundy et al., 2003). Results in Figure 4B showed that more than half of the genes encoding the enzymes which are regulating lysine biosynthesis were up-regulated, and they were mainly in the early stages involved in this pathway. LysA [EC:4.1.1.20] is a decarboxylase enzyme to catalyze meso-2,6-diaminopimelate into L-lysine and the fold change of its encoded gene is + 1.437 (slightly below the baseline of this analysis, and |fold change| <1.50003 will not be highlighted). These results suggested that the lysine biosynthesis of *S. aureus* 29213 has increased under the stress of LCEO.

**FIGURE 5:**
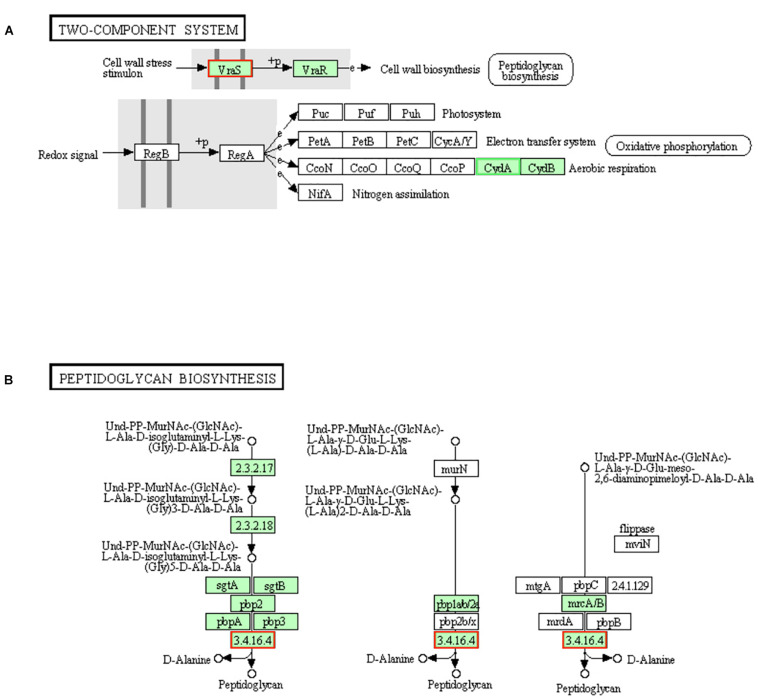
Significantly enriched KEGG pathway “Two-component system” **(A)** and “Peptidoglycan biosynthesis” **(B)** (from KEGG database, green rectangles represent proved Organism-specific gene product and green frames represent down-regulation and red frames represent up-regulation).

The gene (*vraS*) encoding the sensor histidine kinase VraS in two-component system was up-regulated in LCEO treated *S. aureus* 29213 (Figure 5A). Once VraS is stimulated by cell wall stress stimulon, it phosphorylates the histidine residues of itself into VraR, then VraR sends signals to downstream for peptidoglycan biosynthesis (Kuroda et al., 2003; Muthaiyan et al., 2008). So, LCEO is a VraSR-dependent cell wall stress stimulon.

Gene encoding pbp4 [EC:3.4.16.4], one of the enzymes in the final steps of peptidoglycan biosynthesis, was up-regulated by LCEO stress (Figure 5B), which suggests that the biosynthesis of peptidoglycan may be increased. Recent study also showed that there is a close relationship between pbp4 and peptidoglycan fragments (Maya-Martine et al., 2019).

From these results, we can conclude that LCEO is a cell wall-active agent (Hu et al., 2019), and the stress response of LCEO treated *S. aureus* 29213 could be explained by the transcriptional profiling evidences of “Histidine metabolism,” “Lysine biosynthesis,” “Two-component system,” and “Peptidoglycan biosynthesis” pathways.

### DEGs Involved in “Fatty Acid Degradation” and “Nitrogen Metabolism” Pathways

**FIGURE 6:**
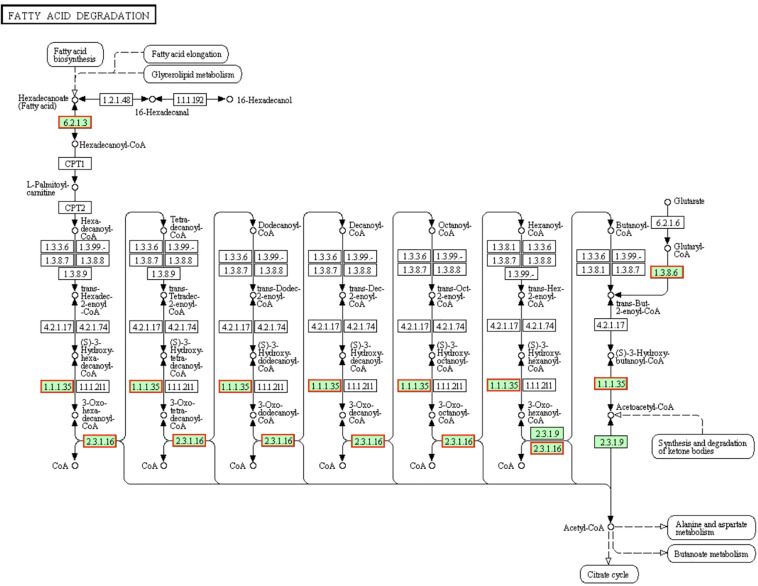
Significantly enriched KEGG pathway “Fatty acid degradation” (from KEGG database, green rectangles represent proved Organism-specific gene product and green frames represent down-regulation and red frames represent up-regulation).

Fatty acids are required to maintain cell viability and *S. aureus* ingest exogenous fatty acids into its phospholipid bilayer (Delekta et al., 2018). The fatty acids in the cytoplasm are synthesized by fadE [EC:6.2.1.3] into hexadecanoyl-CoA then transported to the mitochondria by carnitine palmitoyl transferase I (CPT1) and CPT2. Hexadecanoyl-CoA will be degraded into acetyl-CoA after seven cycles. Each cycles is consists of four steps (dehydrogenation, hydration, dehydrogenation and transformation) and then total 4 mol ATP will be generated by the 2 dehydrogenation processes in a cycle (Parekh, 1977). In present results (Figure 6), genes involved in fatty acid degradation pathway and encoding proteins (fadA [EC:2.3.1.16], fadB [EC:1.1.1.35], fadE [EC:6.2.1.3] and fadD [EC:1.3.8.6]) were up-regulated, which indicated the fatty acid degradation activity of LCEO treated *S. aureus* 29213 was increased. Research showed that the hexose monophophate pathway (HMP) and its key enzyme (glucose-6-phosphate dehydrogenase) activity of methicillin-resistant *S. aureus* were inhibited by LCEO treatment (Hu et al., 2019). However, the |fold change| of genes involved in HMP is under 1.5 in present results. That may be because our *S. aureus* 29213 was treated with LCEO for a short time (only 15 min). Although there was no significantly enriched, present data showed that almost all the screened genes involved in fructose and mannose metabolism, starch and sucrose metabolism, glycolysis/gluconeogenesis and citrate (TCA) cycle were down-regulated (Supplementary Tables 1, 3).

**FIGURE 7:**
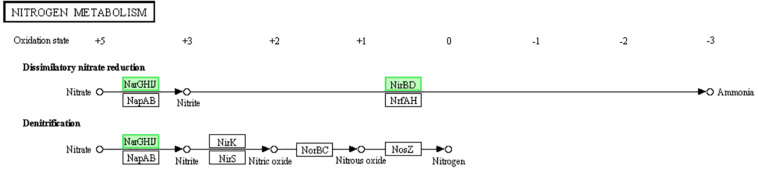
Significantly enriched KEGG pathway “Nitrogen metabolism” (from KEGG database, green rectangles represent proved Organism-specific gene product and green frames represent down-regulation and red frames represent up-regulation).

The existence of dissimilatory nitrate reduction makes the microbes with multiple environmental adaptabilities (such as the metabolism in both oxic and anoxic status) and resource independence (like anaerobic metabolism under indirect nitrate supplyment) (Kamp et al., 2015). Denitrification is a type of respiration. When bacteria are exposed to low or poor oxygen environment, nitrate or nitrite is reduced as a terminal electron acceptor in this respiration (Cabello et al., 2004). Both of these methods can also provide energy to microorganisms (higher than that of fermentation but lower than that of aerobic respiration). The reductases NarGHIJ can reduce nitrate into nitrite, and the reductases NirBD can reduce nitrite into ammonia. As the genes encoding NarGHIJ and NirBD were donw-regulated (Figure 7), we assumed that LCEO inhibits the nitrate respiration function of *S. aureus* 29213. Related studies have shown that the nitrogen metabolism is closely related to the antibiotic resistance (Borriello et al., 2004; Yan et al., 2013). Therefore, we assumed that the combination of LCEO and antibiotics can reduce the use of antibiotics on *S. aureus*.

From this part, we assumed that LCEO inhibited the metabolism of carbohydrate and nitrogen, so that in order to survive, the bacteria has to increase activity of fatty acid degradation.

### DEGs Involved in “Carotenoid Biosynthesis” and “Two-Component System” Pathways

**FIGURE 8:**
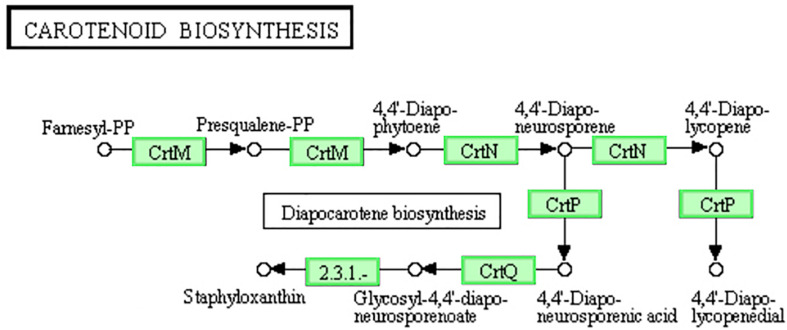
Significantly enriched KEGG pathway “Carotenoid biosynthesis” (from KEGG database, green rectangles represent proved Organism-specific gene product and green frames represent down-regulation and red frames represent up-regulation).

**FIGURE 9:**
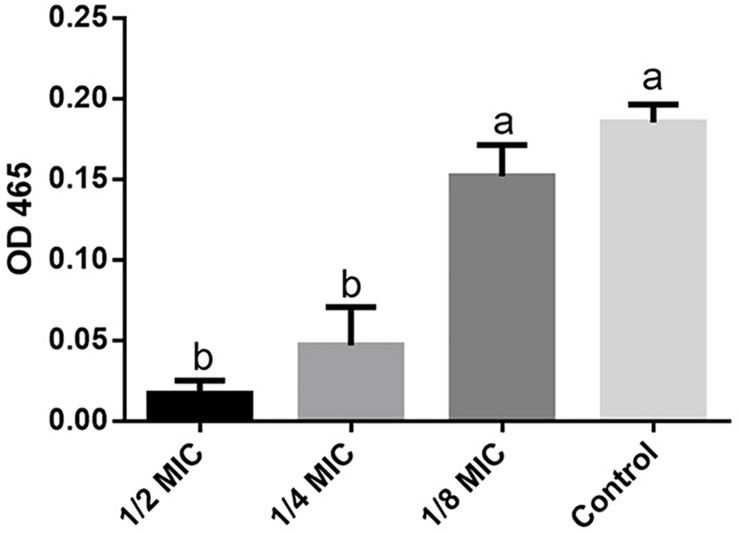
Staphyloxanthin level assay. OD465 value represent the level of staphyloxanthin production in different groups. The data represent the means ± standard deviation of the results. Lowercase marked without the same superscripts (a,b) differ significantly (*P* < 0.05).

Staphyloxanthin is a golden pigment of *S. aureus* and it distinguishes *S. aureus* from other *Staphylococci* even gram-positive cocci. Staphyloxanthin is biosynthesized by a series of steps and is the only C_30_ golden carotenoid of membrane-embedded. Staphyloxanthin is an important antioxidant as well as an anti-host neutrophil-based killing substance. Also, the biosynthesis of staphyloxanthin is under the oxygen-sensing and redox-signaling regulation of the two-component system (Gao et al., 2017; Hall et al., 2017). CrtM and CrtN are the key enzymes of the golden staphyloxanthin biosynthetic process and they could be important targets of novel drug design to handle the infections of *S. aureus* (Yang et al., 2019). According to the results of transcriptome analysis, all the DEGs involved in carotenoid biosynthesis pathway were down-regulated (Figure 8). Furthermore, the down-regulation of *CydA* which is involved in the redox related part of two-component system pathway as well as relevant to aerobic respiration was also recorded (Figure 5A). To further confirm the transcriptomic reports of present analysis, we treated *S. aureus* 29213 strains with LCEO and found that LCEO inhibited the staphyloxanthin lever of *S. aureus* 29213 in a dose-dependent manner (Figure 9). As staphyloxanthin is related to the antioxidant activity of LCEO, this result suggests that *S. aureus* may be more vulnerable to antibacterial agent or environment after treated with LCEO (Wang et al., 2012; Bi et al., 2017; She et al., 2019). If it is true, LCOE will have a broader application prospect.

### DEGs Involved in “*Staphylococcus aureus* Infection” Pathway

*S. aureus* has ability to colonize to the host and to form biofilm, which is likely to be partially determined by its ability of adhesion (Foulston et al., 2014; Scherr et al., 2014). Vitro research showed that the proteins ClfB, IsdA, SdrC/D, and SasG are able to promote adhesion of *staphylococci* (Foster, 2009). Related studies have shown that the formation of biofilm of *S. aureus* is closely related to the genes which encoding these proteins (O’Brien et al., 2002; Feuillie et al., 2017; Liu et al., 2018). Recent report has shown that 1 mg/mL was the minimal biofilm inhibitory concentration of LCEO on *S. aureus* and the MIC is 0.5 mg/mL (Mei, 2019). Thus, the up-regulated genes encoding surface proteins ClfB and SdrC/D (data not shown) which promote the adhesion of *S. aureus* suggested the enhanced biofilm viability to adapt to the unsuitable conditions.

### DEGs Involved in “Pyruvate Metabolism” Pathways

**FIGURE 10:**
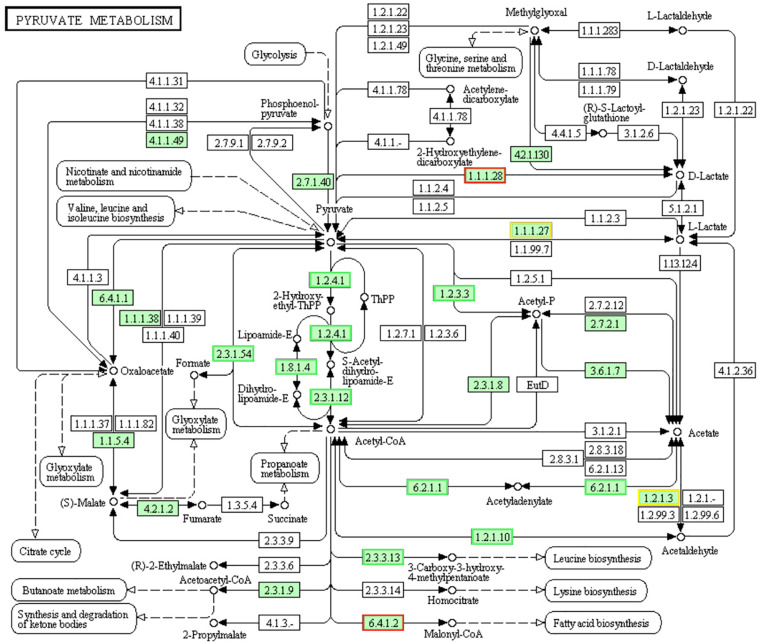
Significantly enriched KEGG pathway “Pyruvate metabolism” (from KEGG database, green rectangles represent proved Organism-specific gene product and green frames represent down-regulation and red frames represent up-regulation, yellow frames represent both down and up-regulation).

Pyruvate is the final product of glycolysis pathway, which is reduced to lactic acid for energy supply in the cytoplasm, or oxidized to acetyl CoA in the mitochondria, and then oxidized to carbon dioxide and water to complete the aerobic energy supply process of glucose. Pyruvate can also convert into sugars, fats and amino acids through the acetyl CoA and tricarboxylic acid cycles. As a consequence, pyruvate plays an important pivotal role in the metabolism of the three major nutrients (Hills, 1938; Sevag et al., 1950). Pyruvate dehydrogenase complex (PDHC) is a group of speed-limiting enzymes that catalyzes the irreversible oxidative decarboxylation of pyruvate into acetyl-CoA. In LCEO treated *S. aureus* 29213, the genes encoding PDHC which consists of pyruvate dehydrogenase E1 component alpha subunit [EC:1.2.4.1], pyruvate dehydrogenase E2 component (dihydrolipoamide acetyltransferase) [EC:2.3.1.12] and dihydrolipoamide dehydrogenase: subunit E3[EC:1.8.1.4] were down-regulated compared with the control (Figure 10). The results may suggest the reduced lever of acetyl-CoA which comes from pyruvate of LCEO treated *S. aureus* 29213. In addition, this may be related to the inhibition effect of carbohydrate metabolism mentioned above.

### RT-PCR Validation

**FIGURE 11:**
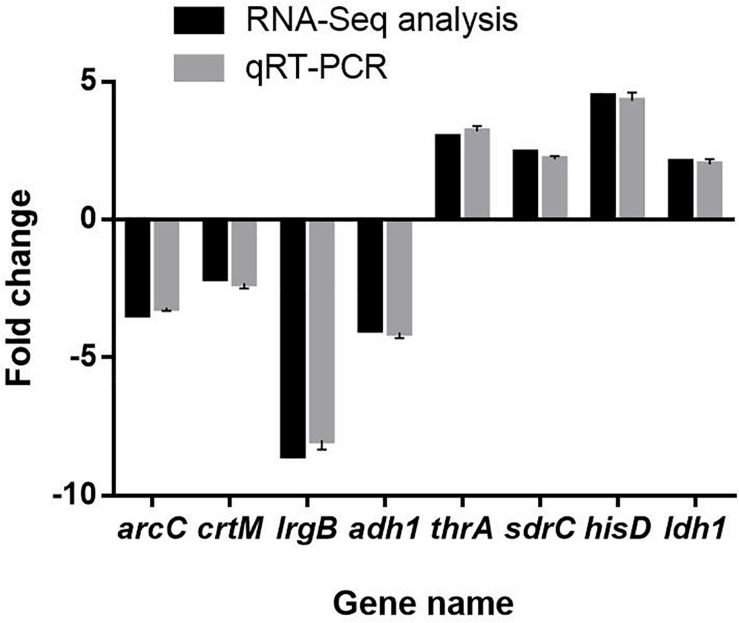
Validation of RNA sequencing data by quantitative real-time PCR. The data represent the means ± standard deviation of the results.

Results of qRT-PCR assay showed that the mRNA expression levels of 8 selected DEGs were highly consistent with the results of RNA-Seq analysis (Figure 11). This result indicated that RNA-Seq assay was properly performed.

## Conclusion

Evidences of genetic lever for the stress response of *S. aureus* 29213 strain under sub-lethal concentration of LCEO by transcriptomic analysis were founded in this study. 300 and 242 genes were significantly up and down-regulated in LCEO-treated *S. aureus* 29213. Up-regulated genes were mainly involved in cell membrane and down-regulated genes were mainly involved in oxidation-reduction process. These findings indicated that the cell membrane stress of *S. aureus* 29213 was occurred by LCEO. At the same time, we reported that LCEO can significantly affect the staphyloxanthin synthesis of *S. aureus* 29213 for the first time, which was closely related to the redox state of *S. aureus* 29213. LCEO may also inhibit the nitrogen metabolism and promote fatty acid degradation of *S. aureus* 29213. These evidences expanded the knowledge of stress response of *S. aureus* 29213 strain under sub-lethal concentration of LCEO.

## Data Availability Statement

The raw data of this manuscript have been deposited in the Genome Sequence Archive (Genomics, Proteomics & Bioinformatics 2017) in BIG Data Center (Nucleic Acids Res 2019), Beijing Institute of Genomics (BIG), Chinese Academy of Sciences, under accession number CRA002405 that is publicly accessible at https://bigd.big.ac.cn/gsa.

## Author Contributions

WZ and HS designed this work. All authors reviewed and approved the final manuscript.

## Conflict of Interest

The authors declare that the research was conducted in the absence of any commercial or financial relationships that could be construed as a potential conflict of interest.

## References

[B1] Argudin M. A. Mendoza M. C. Rodicio M. R. (2010). Food poisoning and *Staphylococcus aureus* enterotoxins. *Toxins* 2 1751–1773. 10.3390/toxins2071751 22069659 PMC3153270

[B2] Bi Y. G. Zhang Y. F. Kong F. S. (2017). “Experimental study on scavenging DPPH free radical activity of litsea cubeba oil,” in *Proceedings of the 2017 7th International Conference on Mechatronics, Computer And Education Informationization (Mcei 2017)* (Paris: Atlantis Press) Vol. 75 939–942.

[B3] Borriello G. Werner E. Roe F. Kim A. M. Ehrlich G. D. Stewart P. S. (2004). Oxygen limitation contributes to antibiotic tolerance of *Pseudomonas aeruginosa* in biofilms. *Antimicrob. Agents Chemother.* 48 2659–2664. 10.1128/Aac.48.7.2659-2664.2004 15215123 PMC434183

[B4] Cabello P. Roldan M. D. Moreno-Vivian C. (2004). Nitrate reduction and the nitrogen cycle in archaea. *Microbiology* 150 3527–3546. 10.1099/mic.0.27303-27300 15528644

[B5] Chen Y. Wang Y. Han X. Si L. Wu Q. Lin L. (2013). Biology and chemistry of Litsea cubeba, a promising industrial tree in China. *J. Essent. Oil Res.* 25 103–111. 10.1080/10412905.2012.751559

[B6] Delekta P. C. Shook J. C. Lydic T. A. Mulks M. H. Hammer N. D. (2018). *Staphylococcus aureus* utilizes host-derived lipoprotein particles as sources of fatty acids. *J. Bacteriol.* 200:e00728-17 10.1128/JB.00728-717 PMC5952394 29581406

[B7] Delpech P. Bornes S. Alaterre E. Bonnet M. Gagne G. Montel M. C. (2015). *Staphylococcus aureus* transcriptomic response to inhibition by H2O2-producing *Lactococcus garvieae*. *Food Microbiol.* 51 163–170. 10.1016/j.fm.2015.05.014 26187841

[B8] Feuillie C. Formosa-Dague C. Hays L. M. Vervaeck O. Derclaye S. Brennan M. P. (2017). Molecular interactions and inhibition of the staphylococcal biofilm-forming protein SdrC. *Proc. Natl. Acad. Sci. U.S.A.* 114 3738–3743. 10.1073/pnas.1616805114 28320940 PMC5389287

[B9] Foster T. J. (2009). Colonization and infection of the human host by staphylococci: adhesion, survival and immune evasion. *Vet. Dermatol.* 20 456–470. 10.1111/j.1365-3164.2009.00825.x 20178484

[B10] Foulston L. Elsholz A. K. W. DeFrancesco A. S. Losick R. (2014). The extracellular matrix of *Staphylococcus aureus* biofilms comprises cytoplasmic proteins that associate with the cell surface in response to decreasing pH. *Mbio* 5:e01667-14 10.1128/mBio.01667-1614 PMC4173787 25182325

[B11] Gao P. Davies J. Kao R. Y. T. (2017). Dehydrosqualene desaturase as a novel target for anti-virulence therapy against *Staphylococcus aureus*. *mBio* 8:e1224-17 10.1128/mBio.01224-1217 PMC5587911 28874472

[B12] Grundy F. J. Lehman S. C. Henkin T. M. (2003). The L box regulon: lysine sensing by leader RNAs of bacterial lysine biosynthesis genes. *Proc. Natl. Acad. Sci. U.S.A.* 100 12057–12062. 10.1073/pnas.2133705100 14523230 PMC218712

[B13] Hall J. W. Yang J. Guo H. Ji Y. (2017). The *Staphylococcus aureus* AirSR two-component system mediates reactive oxygen species resistance via transcriptional regulation of staphyloxanthin production. *Infect. Immun.* 85:e00838-16 10.1128/IAI.00838-816 PMC5278164 27872240

[B14] Harms N. Reijnders W. N. Koning S. van Spanning R. J. (2001). Two-component system that regulates methanol and formaldehyde oxidation in *Paracoccus* denitrificans. *J. Bacteriol.* 183 664–670. 10.1128/JB.183.2.664-670.2001 11133961 PMC94923

[B15] Henriksen S. T. Liu J. Estiu G. Oltvai Z. N. Wiest O. (2011). Identification of novel bacterial histidine biosynthesis inhibitors using docking, ensemble rescoring, and whole-cell assays. *Bioorg. Med. Chem.* 18 5148–5156. 10.1016/j.bmc.2010.05.060 20573514 PMC2903657

[B16] Hills G. M. (1938). Aneurin (vitamin B(1)) and pyruvate metabolism by *Staphylococcus aureus*. *Biochem. J.* 32 383–391. 10.1042/bj0320383 16746631 PMC1264037

[B17] Ho C. L. Jie-Pinge O. Liu Y. C. Hung C. P. Tsai M. C. Liao P. C. (2010). Compositions and in vitro anticancer activities of the leaf and fruit oils of *Litsea cubeba* from Taiwan. *Nat. Prod. Commun.* 5 617–620. 20433084

[B18] Hu W. Li C. Z. Dai J. M. Cui H. Y. Lin L. (2019). Antibacterial activity and mechanism of *Litsea cubeba* essential oil against methicillin-resistant *Staphylococcus aureus* (MRSA). *Indust. Crops Prod.* 130 34–41. 10.1016/j.indcrop.2018.12.078

[B19] Hwang J. K. Choi E. M. Lee J. H. (2005). Antioxidant activity of *Litsea cubeba*. *Fitoterapia* 76 684–686. 10.1016/j.fitote.2005.05.007 16239077

[B20] Kamp A. Hogslund S. Risgaard-Petersen N. Stief P. (2015). Nitrate storage and dissimilatory nitrate reduction by eukaryotic microbes. *Front. Microbiol.* 6:1492. 10.3389/fmicb.2015.01492 26734001 PMC4686598

[B21] Kuroda M. Kuroda H. Oshima T. Takeuchi F. Mori H. Hiramatsu K. (2003). Two-component system VraSR positively modulates the regulation of cell-wall biosynthesis pathway in *Staphylococcus aureus*. *Mol. Microbiol.* 49 807–821. 10.1046/j.1365-2958.2003.03599.x 12864861

[B22] Liao P. C. Yang T. S. Chou J. C. Chen J. Lee S. C. Kuo Y. H. (2015). Anti-inflammatory activity of neral and geranial isolated from fruits of *Litsea cubeba* Lour. *J. Funct. Foods* 19 248–258. 10.1016/j.jff.2015.09.034

[B23] Liu G. F. Su H. Z. Sun H. Y. Lu G. T. Tang J. L. (2019). Competitive control of endoglucanase gene engXCA expression in the plant pathogen *Xanthomonas campestris* by the global transcriptional regulators HpaR1 and Clp. *Mol. Plant Pathol.* 20 51–68. 10.1111/mpp.12739 30091270 PMC6430473

[B24] Liu J. Y. Yang L. Hou Y. C. Soteyome T. Zeng B. B. Su J. Y. (2018). Transcriptomics Study on *Staphylococcus aureus* biofilm under low concentration of ampicillin. *Front. Microbiol.* 9:2413. 10.3389/Fmicb.2018.02413 30425687 PMC6218852

[B25] Liu T. T. Yang T. S. (2012). Antimicrobial impact of the components of essential oil of Litsea cubeba from Taiwan and antimicrobial activity of the oil in food systems. *Int. J. Food Microbiol.* 156 68–75. 10.1016/j.ijfoodmicro.2012.03.005 22459760

[B26] Lowy F. D. (1998). *Staphylococcus aureus* infections. *N. Engl. J. Med.* 339 520–532. 10.1056/NEJM199808203390806 9709046

[B27] Maya-Martine R. Alexander J. A. N. Otten C. F. Ayala I. Vollmer D. Gray J. (2019). Recognition of peptidoglycan fragments by the transpeptidase PBP4 From *Staphylococcus aureus*. *Front. Microbiol.* 9:3223. 10.3389/Fmicb.2018.03223 30713527 PMC6346638

[B28] Mei B. (2019). *Inhibition Mechanism of Litsea cubeba Essential oil on Staphylococcus aureus and its Biofilm*, Master’s thesis, Jiangsu University, China.

[B29] Musthafa K. S. Voravuthikunchai S. P. (2015). Anti-virulence potential of eugenyl acetate against pathogenic bacteria of medical importance. *Antonie Van Leeuwenhoek* 107 703–710. 10.1007/s10482-014-0364-364 25613850

[B30] Muthaiyan A. Silverman J. A. Jayaswal R. K. Wilkinson B. J. (2008). Transcriptional profiling reveals that daptomycin induces the *Staphylococcus aureus* cell wall stress stimulon and genes responsive to membrane depolarization. *Antimicrob. Agents Chemother.* 52 980–990. 10.1128/Aac.01121-1127 18086846 PMC2258546

[B31] Nguyen H. V. Caruso D. Lebrun M. Nguyen N. T. Trinh T. T. Meile J. C. (2016). Antibacterial activity of *Litsea cubeba* (*Lauraceae*, May Chang) and its effects on the biological response of common carp *Cyprinus carpio* challenged with *Aeromonas hydrophila*. *J. Appl. Microbiol.* 121 341–351. 27124660 10.1111/jam.13160

[B32] O’Brien L. M. Walsh E. J. Massey R. C. Peacock S. J. Foster T. J. (2002). Staphylococcus aureus clumping factor B (ClfB) promotes adherence to human type I cytokeratin 10: implications for nasal colonization. *Cell Microbiol.* 4 759–770. 10.1046/j.1462-5822.2002.00231.x 12427098

[B33] Parekh V. R. (1977). N-Alkane oxidation enzymes of a Pseudomonad. *Appl. Environ. Microbiol.* 33 881–884. 10.1128/aem.33.4.881-884.1977 869535 PMC170785

[B34] Park J. Y. Jo S. K. Park K. M. Yu H. Bai J. Ryu S. (2019). Transcriptomic analysis of *Staphylococcus aureus* under the stress condition of antibacterial erythorbyl laurate by RNA sequencing. *Food Control* 96 1–8. 10.1016/j.foodcont.2018.08.021

[B35] Qiao Q. Huang Y. Y. Qi J. Qu M. Z. Jiang C. Lin P. C. (2016). The genome and transcriptome of Trichormus sp NMC-1: insights into adaptation to extreme environments on the Qinghai-Tibet Plateau. *Sci. Rep.* 6:29404. 10.1038/Srep29404 27381465 PMC4933973

[B36] Sadrearhami Z. Shafiee F. N. Ho K. K. K. Kumar N. Krasowska M. Blencowe A. (2019). Antibiofilm nitric oxide-releasing polydopamine coatings. *ACS Appl. Mater Interfaces* 11 7320–7329. 10.1021/acsami.8b16853 30688429

[B37] Scherr T. D. Heim C. E. Morrison J. M. Kielian T. (2014). Hiding in plain sight: interplay between staphylococcal biofilms and host immunity. *Front. Immunol.* 5:37. 10.3389/Fimmu.2014.00037 24550921 PMC3913997

[B38] Seo S. M. Kim J. Lee S. G. Shin C. H. Shin S. C. Park I. K. (2009). Fumigant antitermitic activity of plant essential oils and components from Ajowan (*Trachyspermum ammi*), Allspice (*Pimenta dioica*), caraway (*Carum carvi*), dill (*Anethum graveolens*), Geranium (*Pelargonium graveolens*), and Litsea (*Litsea cubeba*) oils against Japanese termite (*Reticulitermes speratus* Kolbe). *J. Agric. Food Chem.* 57 6596–6602. 10.1021/jf9015416 19722567

[B39] Sevag M. G. Steers E. Forbes M. (1950). The mechanism of resistance of sulfonamides; a comparative study of the resistance to sulfathiazole of the metabolism of glucose and pyruvate by *Staphylococcus aureus*. *Arch. Biochem.* 25 185–190. 15401228

[B40] She Q. H. Li W. S. Jiang Y. Y. Wu Y. C. Zhou Y. H. Zhang L. (2019). Chemical composition, antimicrobial activity and antioxidant activity of *Litsea cubeba* essential oils in different months. *Nat. Prod. Res.* 10.1080/14786419.2018.1557177 Online ahead of print 30931646

[B41] Si L. Chen Y. Han X. Zhan Z. Tian S. Cui Q. (2012). Chemical composition of essential oils of Litsea cubeba harvested from its distribution areas in China. *Molecules* 17 7057–7066. 10.3390/molecules17067057 22683894 PMC6268156

[B42] Singh N. Rajwade J. Paknikar K. M. (2019). Transcriptome analysis of silver nanoparticles treated *Staphylococcus aureus* reveals potential targets for biofilm inhibition. *Colloids Surf. B Biointerfaces* 175 487–497. 10.1016/j.colsurfb.2018.12.032 30572157

[B43] Singh V. K. Sirobhushanam S. Ring R. P. Singh S. Gatto C. Wilkinson B. J. (2018). Roles of pyruvate dehydrogenase and branched-chain alpha-keto acid dehydrogenase in branched-chain membrane fatty acid levels and associated functions in *Staphylococcus aureus*. *J. Med. Microbiol.* 67 570–578. 10.1099/jmm.0.000707 29498620 PMC5982145

[B44] Wang H. Liu Y. (2010). Chemical composition and antibacterial activity of essential oils from different parts of *Litsea cubeba*. *Chem. Biodivers.* 7 229–235. 10.1002/cbdv.200800349 20087994

[B45] Wang Y. Jiang Z. T. Li R. (2009). Complexation and molecular microcapsules of Litsea cubeba essential oil with beta-cyclodextrin and its derivatives. *Eur. Food Res. Technol.* 228 865–873. 10.1007/s00217-008-0999-993

[B46] Wang Y. Jiang Z. T. Li R. (2012). Antioxidant activity, free radical scavenging potential and chemical composition of *Litsea cubeba* essential oil. *J. Essent. Oil Bearing Plants* 15 134–143. 10.1080/0972060X.2012.10644029

[B47] Wolanin P. M. Thomason P. A. Stock J. B. (2002). Histidine protein kinases: key signal transducers outside the animal kingdom. *Genome Biol.* 3:REVIEWS3013. 10.1186/gb-2002-3-10-reviews3013 12372152 PMC244915

[B48] Yan C. Dinh Q. T. Chevreuil M. Garnier J. Roose-Amsaleg C. Labadie P. (2013). The effect of environmental and therapeutic concentrations of antibiotics on nitrate reduction rates in river sediment. *Water Res.* 47 3654–3662. 10.1016/j.watres.2013.04.025 23726701

[B49] Yang Y. Guo L. Tariq K. Zhang W. Li C. Memon F. Q. (2020). The antioxidant polysaccharide from *Semiaquilegia adoxoides* (DC.) makino adjusts the immune response of mice infected by bacteria. *Evid. Based Complement Alternat. Med.* 2020:2719483. 10.1155/2020/2719483 32148535 PMC7049844

[B50] Yang Y. Wang H. Zhou H. Hu Z. Shang W. Rao Y. (2019). Protectiveeffect of the golden staphyloxanthin biosynthetic pathway on *Staphylococcus aureus* under cold atmospheric plasma treatment. *Appl. Environ. Microbiol.* 86 1–9. 10.1128/AEM.01998-1919 PMC6974630 31704682

[B51] Zhili J. Yasmin A. Rod B. Xing Z. Isman M. B. (2009). Comparative toxicity of essential oils of *Litsea pungens* and *Litsea cubeba* and blends of their major constituents against the cabbage looper, Trichoplusia ni. *J. Agric. Food Chem.* 57 4833–4837. 10.1021/jf900274r 19422220

