# Supplementary figures and images for: Transcriptomic Analysis of Staphylococcus aureus Under the Stress Condition Caused by Litsea cubeba L. Essential Oil via RNA Sequencing
Source: Front Microbiol. 2020 Sep 8;11:1693. doi: 10.3389/fmicb.2020.01693 (PMC7509438; doi:10.3389/fmicb.2020.01693)

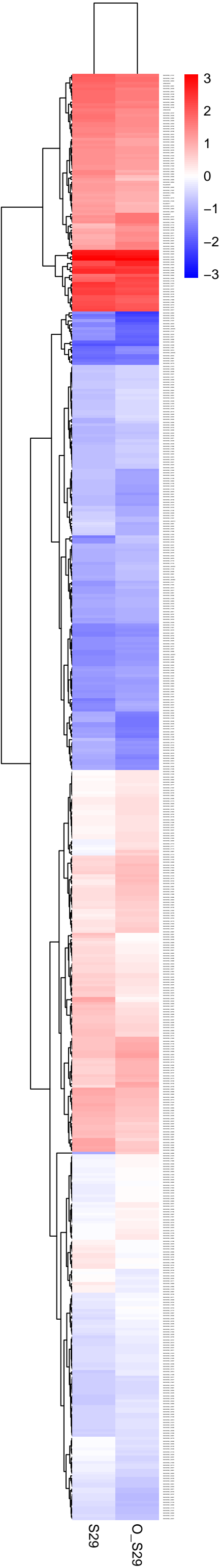

O\_S29  
S29

Supplement: Supplementary file 1 [file Data_Sheet_1.PDF]
